# Dual-Frequency Ultrasound Enhances Cavitation of Microdroplets for Controlled Scaffold Porosity in Tissue Engineering

**DOI:** 10.1021/acsami.5c19957

**Published:** 2026-04-30

**Authors:** Hen Shenhav, Bar Glickstein, Tiran Bercovici, Offir Loboda, Gal Shklarski Shchori, Dekel Rosenfeld, Lihi Adler-Abramovich, Tali Ilovitsh

**Affiliations:** † School of Biomedical Engineering, Iby and Aladar Fleischman Faculty of Engineering, 26745 Tel Aviv University, Tel Aviv 6997801, Israel; ‡ Department of Oral Biology, the Goldschleger School of Dental Medicine, Gray Faculty of Medical & Health Sciences, Tel Aviv University, Tel Aviv 6997801, Israel; § Jan Koum Center for Nanoscience and Nanotechnology, Tel Aviv University, Tel Aviv 6997801, Israel; ∥ The Center for Physics & Chemistry of Living Systems, Tel Aviv University, Tel Aviv 6997801, Israel; ⊥ Department of Materials Science and Engineering, Iby and Aladar Fleischman Faculty of Engineering, Tel Aviv University, Tel Aviv 6997801, Israel; # The Sagol School of Neuroscience, Tel Aviv University, Tel Aviv 6997801, Israel

**Keywords:** focused ultrasound, tissue engineering, low frequency, microdroplets, acoustic droplet vaporization

## Abstract

The development of porous scaffolds with tunable mechanical and structural properties is essential for advancing tissue engineering strategies. In this study, we present a noninvasive, adjustable method for generating porous collagen scaffolds by utilizing micron-sized phase-shift droplets in combination with dual-frequency ultrasound. These microdroplets, generated via a microfluidic chip and composed of a liquid perfluoropentane core stabilized by a phospholipid shell, were embedded within collagen hydrogels and served as ultrasound-responsive cavitation nuclei. A 3.5 MHz imaging transducer was employed to trigger acoustic droplet vaporization of the embedded microdroplets, transitioning them into microbubbles. Then, a 200 kHz therapeutic transducer induced bubble oscillation and collapse, leading to localized pore formation. This combined ultrasound strategy enabled both vaporization and bubble implosion at reduced pressure thresholds compared to conventional acoustic droplet vaporization methods. Theoretical modeling using the Marmottant model predicted microbubble dynamics and corresponding pore sizes, which were validated through scanning electron microscopy and histological analysis. Ultrasound-treated scaffolds containing droplets exhibited significantly increased porosity of 56.53 ± 3.91% compared to untreated controls, with a pore diameter of 39.42 ± 10.28 μm, observed via scanning electron microscopy. Rheological analysis revealed enhanced elasticity and structural resilience in ultrasound-treated scaffolds. Finally, in vitro studies confirmed that fibroblast viability remained high within the treated scaffolds, with cells observed in close proximity to ultrasound-generated pores. This work introduces a tunable and clinically relevant strategy for fabricating functional scaffolds that could support tissue regeneration and customizable healing environments.

## Graphical abstract

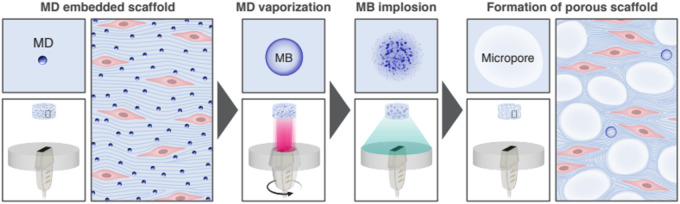

## Introduction

Diseases, injuries, and traumatic events often result in tissue damage and degeneration, thereby necessitating therapeutic strategies aimed at repair, replacement, or regeneration.^1^ Tissue engineering addresses this need by seeking to restore, maintain, or enhance compromised tissue function, either through the development of biological substitutes or by reconstructing damaged tissues, using natural and synthetic materials.^2,3^ Scaffolds are often used in tissue engineering applications to provide mechanical support, a framework for cells to attach, proliferate, and to mimic the extracellular matrix (ECM).^4,5^ An important scaffold material is collagen, the most abundant protein in the ECM, which plays a pivotal role in preserving the ECM’s biological and structural integrity.^6^ Its inherent properties, such as low immunogenicity, high permeability, excellent biocompatibility, and biodegradability, render collagen a highly promising biomaterial for scaffold fabrication in tissue regeneration applications. However, non-cross-linked collagen scaffolds often exhibit limited mechanical strength and structural stability, which can restrict their use in load-bearing tissues.^7–9^ Porosity in scaffolds is essential for new tissue formation, as it facilitates three-dimensional (3D) cell nutrition, migration and proliferation.^10–12^ In addition, the porosity also allows for tissue vascularization, oxygen transport, and waste removal, which are critical factors for the generation of tissues.^12,13^ However, increased scaffold porosity often compromises mechanical properties, which are essential for maintaining structural stability and to support the tissue during regeneration.^12,14^

Several techniques are used to introduce porosity in scaffolds for tissue engineering. Solvent casting enables precise control over pore size but is limited to thin scaffolds, restricting its applicability, and may leave behind residual particles that can negatively affect cell viability.^15–17^ Thermally induced phase separation offers adjustable pore structures through processing parameter control, yet it often produces micropores that are too small to support adequate cell infiltration.^16^ Freeze-drying is another commonly used method that allows customization of porosity and pore size, although it tends to generate irregular pore architectures.^15^ Moreover, those methods modify mechanical and structural properties instantly and uniformly, lacking the ability to provide spatiotemporal control when changes are needed at different times. To address these limitations, we propose an approach for generating tunable porous collagen scaffolds using ultrasound (US) and micron-sized phase-shift droplets (MDs) as cavitation nuclei. Microbubbles (MBs), commonly used as US contrast agents, typically measure between 1 and 10 μm in diameter. They are composed of a phospholipid shell encapsulating a perfluorocarbon (PFC) core, which enhances their stability in the blood. Under US exposure at imaging frequencies (2–10 MHz) and low acoustic peak negative pressures (PNP), MBs exhibit stable, symmetric oscillations, a phenomenon known as stable cavitation. When subjected to higher acoustic PNPs, their oscillations enhance until resulting in a violent collapse referred to as inertial cavitation (IC). This process releases significant mechanical energy, which can generate localized effects in the surrounding medium.^18–20^ We previously showed that insonation at lower US frequencies (below 250 kHz) enhances MB oscillations, and we utilized this property to induce mechanical tumor fractionation and sonoporation at low PNP.^21–25^ Here, we hypothesize that low-frequency US, combined with contrast agents, can facilitate large pore formation for tissue engineering applications. However, both incubation at physiological temperature and circulation in vivo rapidly destabilize MBs, restricting their stability to only a few minutes under these conditions,^26^ which makes them less suitable for applications requiring extended incubation.

For effective tissue engineering, sustained stability is necessary to ensure accurate timing in the modulation of scaffold porosity. Therefore, phase-shift PFC droplets have been proposed as an improved alternative to MBs, offering greater stability, controllable vaporization, and serving as effective cavitation nuclei. These droplets function as US contrast agents and contain a liquid PFC core at room temperature. Upon exposure to US, the liquid core vaporizes into gas, forming MBs, a process known as acoustic droplet vaporization (ADV).^27,28^ Similar to MBs, increasing the applied PNP in the process can lead to IC of the vaporized droplets. Here, this process will be harnessed for the formation of porous scaffolds.

Droplets can be generated via multiple techniques, including condensation, where a PFC gas core is liquefied by applying pressure alongside temperature reduction. Previously, we used perfluorobutane nanodroplets for US mechanotherapy of tumors.^29–31^ However, due to the low boiling point of perfluorobutane (−2 °C), these droplets are not thermally stable enough for tissue engineering applications. In addition, their diameter (∼300 nm) is too small to generate pores in the tens-of-micrometers range required for effective scaffold fabrication. An alternative approach is to use microfluidics for MD fabrication, where the final particle diameter is regulated by total flow rate, flow rate ratio, and channel geometry. In this approach, we use higher-order PFCs with higher boiling points that remain liquid at room temperature, thus forming thermally stable MDs. US, in combination with MDs, has previously been employed to create porous fibrin scaffolds for tissue engineering. However, those MDs were based on Pluronic shells, which are generally less stable than lipid-based shells.^32^ Importantly, US was applied at center frequencies of 1.1 and 2.5 MHz with PNPs up to 5.5 MPa, yielding mechanical index (MI) values that exceed the FDA-recommended safety limit of 1.9 for diagnostic imaging.^33^ Although the MI was originally defined for diagnostic US, it remains a relevant translational benchmark, as eventual FDA approval will require a clear safety framework for in vivo application to implanted scaffolds. Operating at MI < 1.9 therefore provides a conservative, widely recognized standard that can facilitate safety justification and clinical translation, even though it is not required for therapeutic efficacy.

The combination of US with MDs embedded in scaffolds has been demonstrated in other applications, such as US-triggered release of growth factors from fibrin scaffolds,^34^ and the differentiation of mesenchymal stromal cells within collagen scaffolds through US-induced modulation of scaffold mechanical properties.^35^

Here, we propose a low-pressure approach to create porous scaffolds using low-frequency US. The novelty of our method lies both in the chemical formulation of MDs, which incorporates a phospholipid shell to enhance stability, and in the US treatment protocol, which leverages low-frequency insonation for efficient pore formation. We fabricate perfluoropentane MDs with an average diameter of around 1.1 μm. These droplets require a PNP of approximately 3 MPa to vaporize. At low frequencies, this translates to an MI well above the FDA safety limit of 1.9 for diagnostic imaging. To address this, we developed a two-step activation strategy that ensures vaporization and subsequent cavitation, while maintaining the MI within safe limits (MI ≤ 1.84 at all of the stages). First, an imaging transducer operating in the MHz range vaporizes the MDs into cavitating MBs. During vaporization, rapid volume expansion generates local stresses that compact and rearrange collagen fibrils,^35^ promoting pore formation and enlargement within the scaffold. This is followed by 200 kHz US, which induces MB collapse and localized tissue disruption. Operating at low MI is essential to ensure that porosity is generated only within the target region containing MDs, minimizing unintended effects in surrounding healthy tissue. These MDs were embedded within a collagen hydrogel and exposed to this dual-frequency US protocol to form a tunable, porous scaffold with controllable mechanical properties, suitable for tissue engineering applications (Figure 1).

**Figure 1:**
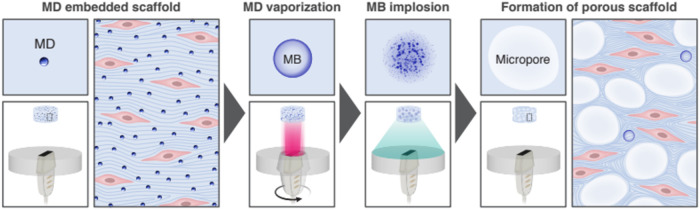
Schematic illustration of the formation of the porous scaffold process. MDs were embedded within the collagen scaffold, followed by US insonation. First, a rotating imaging transducer was used to induce MB formation. Then, a low-frequency therapeutic transducer was applied to generate IC, leading to the formation of a porous scaffold.

## Materials and Methods

### MD Preparation

The MDs used in this study consisted of a phospholipid shell and a perfluoropentane (C_5_F_12,_ PFP) core. The preparation of MDs was conducted in two stages. First, a lipid solution was prepared following the methods described in ref.^21^ Lipid powders (2.5 mg/mL) of distearoylphosphatidylcholine (DSPC) and 1,2-distearoyl-*sn*-glycero-3-phosphoethanolamine-*N*-[methoxy­(polyethylene glycol)-2000] (ammonium salt) (DSPE-PEG2K) were dissolved in 10% propylene glycol (PG, Sigma-Aldrich, Milwaukee, WI) at a molar ratio of 90:10. This lipid mixture was then heated and sonicated at 62 °C. Next, a preheated (62 °C) mixture of 10% glycerol (Gly, Acros Organics) and 80% phosphate-buffered saline (PBS, pH 7.4) was added to the lipid solution, and the combined mixture was sonicated for 10 min at room temperature. In the second stage, the droplet structure was formed using a microfluidic chip (0.2 mm channel width, Herringbone Mixer Glass Chip, Darwin Microfluidics, U.K.) and two syringe pumps (NE-300 InfusionONE Syringe Pump, Darwin Microfluidics, U.K.). The lipid solution was pumped into the chip at a velocity of 600 μm/min, while the PFP was introduced at a velocity of 200 μm/min. To produce fluorescent MDs, the PFP was vortexed with BODIPY (Difluoro­{2-[(3,5-dimethyl-2*H*-pyrrol-2-ylidene-*N*)­methyl]-3,5-dimethyl-1*H*-pyrrolato N}­boron, Sigma- Aldrich, Milwaukee, WI) before MD formation. MDs morphology was examined using an Echo Revolution microscope (ECHO, San Diego, CA, USA). The size distribution and concentration of the MDs were measured using a particle counter system (AccuSizer FX-Nano, Particle Sizing Systems, Entegris, MA, USA). The MDs were stored in 4 °C and their storage stability was evaluated over 0–7 months.

### Ultrasound Setup

The US setup, similar to the one used in ref,^31^ was designed for 3D volumetric vaporization and detonation of the MDs. Briefly, the system consisted of a 1D rotating imaging array controlled by a motorized rotary, positioned within a therapeutic transducer at the bottom of a water tank. The rotation of the imaging array enables exposure from multiple angles, thereby achieving volumetric vaporization. The therapeutic transducer had a focal distance of 65 mm, so the elevation focus of the imaging transducer was set to the same distance. The imaging transducer (IP104, Sonic Concepts, Bothell, WA, USA) has 128 elements with an aperture elevation of 13.5 mm and an azimuthal aperture of 28.2 mm. The transducer was operated using a programmable US system (Vantage 256, Verasonics Inc., Redmond, WA, USA) and was mounted on a motorized rotary system (RTY-IP100, Sonic Concepts). This system, consisting of a rotary motor and a gear mechanism, allowed precise ±180° rotation of the imaging probe relative to the fixed therapeutic transducer. The hermetically sealed design enabled frictionless rotation, which was controlled via MATLAB scripts (version 2021b, MathWorks, Natick, MA). These scripts provided control over the imaging probe’s rotation angle, speed, and acceleration. The rotating imaging transducer was used to vaporize MDs by transmitting a 2-cycle sinusoidal pulse at a center frequency of 3.5 MHz and at PRF of 20 Hz (duty cycle ≈ 1.7%), focused on the target site (*z* = 65 mm). This low duty cycle limits the temporal-average acoustic intensity and minimizes heat buildup. It is well below levels reported in recent studies, where duty cycles up to ∼15% did not cause heat-induced cell death.^36^ Additionally, it captured US images of the MDs before and after each vaporization optimization experiment. For the stability experiment, a perpendicular imaging transducer was used (L7–4, Philips, ATL). The L7–4 transducer is also controlled by the programmable US system. This transducer has 128 elements, with an element size of 7 mm × 0.283 mm (height × width), a kerf width of 0.025 mm and operates at a center frequency of 5 MHz. The low-frequency therapeutic transducer was a spherically focused, single-element transducer (H149, Sonic Concepts) that operates at center frequencies of 105 and 200 kHz via custom matching networks (purchased from Sonic Concepts). In these experiments, a center frequency of 200 kHz was chosen, with lateral and axial full-width half-maximum values of 4.4 mm and 18.5 mm, respectively. The transducer, focused at 65 mm, was driven by a transducer power output unit (TPO-200, Sonic Concepts). Both transducers’ PNPs were calibrated in water using a needle hydrophone (NH0200, Precision Acoustics, U.K.). Agarose and collagen hydrogels exhibit low acoustic attenuation. For the experiments performed in agarose phantom, the 3.5 MHz US propagated through 5 mm of agar, corresponding to an estimated attenuation of ∼0.525 dB.^37^ For the collagen hydrogel, the propagation depth was 3 mm, resulting in an estimated attenuation of 0.036 dB at 3.5 MHz, and 0.00036 dB at 200 kHz.^38^ Given these low attenuation values, the free-field pressure measurements provide a reasonable approximation of the in situ acoustic pressures.

For the stability and vaporization optimization experiments, an agarose phantom containing a diluted MD solution was positioned at the focal point of both transducers. For the vaporization and detonation of the MDs within the collagen scaffold, the scaffolds were placed at the transducers’ focal point.

### Agarose Phantom Preparation

The agarose phantoms were prepared by mixing 1.5% agar powder (A0439758, Thermo Scientific, MA, USA) and deionized water. The solution then heated until the powder was totally dissolved. The heated solution was poured into a custom mold and left to cool. The mold measured 65 mm × 25 mm × 20 mm (length × width × height) and contained a centrally positioned aluminum rod, 15 mm in height and 6 mm in diameter. After being removed from the mold, the phantoms were placed at the focal points of both transducers in the US setup, and the rod-shaped cavity was filled with the MD suspension.

### MD Stability Experiment

The objective of this experiment was to assess the stability of MDs at 37 °C, which corresponds to both physiological temperature and the collagen polymerization temperature.^39^ A suspension containing 4.8 × 10^6^ MDs/ml, diluted in degassed, prewarmed (37 °C) PBS at a 1:100 ratio, and incubated at this temperature for varying durations ranging from 0 to 60 min, in 5 min intervals. At each point, an aliquot of the mixture was injected into the rod inclusion within the agar phantom until the inclusion was completely filled. Subsequently, images were acquired using the L7–4 imaging transducer, operating at a center frequency of 5 MHz. Postprocessing of the captured images was performed to quantify changes in contrast, reflecting the vaporization of MDs into MBs and thereby assessing MD stability. Although the speed of sound in PFP is lower than in water, its acoustic echogenicity is significantly lower than gas MBs. The contrast variation was determined using (eq 1)^40^

1 $$\mathrm{Contrast}\left[\mathrm{dB}\right]=20\log_{10}\frac{\mu_{i}}{\mu_{0}}$$

where μ*_i_* represents the mean pixel intensity within the region of interest (ROI) inside the MD inclusion at a given time point *i*, and μ_0_ corresponds to the mean pixel intensity within the ROI at the initial (zero) time point.

### MD Vaporization Optimization

The aim of these experiments was to optimize the PNP required to induce the vaporization of MDs into MBs and to determine their expansion following vaporization. The rotated imaging transducer enabled the superposition of multiple rotating vaporization lines, forming a circular vaporization pattern, as previously described.^31^ In addition to the vaporization process, the imaging transducer (center frequency of 3.5 MHz) was also utilized for the US image acquisition, both before and after vaporization. The transducer’s rotation speed and acceleration were set to 95°/s and 2000°/s^2^, respectively. A suspension containing 4.8 × 10^6^ MDs/ml, diluted in degassed PBS at a 1:100 ratio, was injected into the rod-shaped inclusion within an agar phantom until the inclusion was completely filled. A two-cycle excitation pulse with a duration of 2 s and PNPs ranging from 2.3 to 3.8 MPa (MI range: 1.2–2) was then applied to the MD inclusions to induce vaporization, transitioning the PFP core from the liquid to the gas phase. The contrast variation was quantified using (eq 1). Here, μ*_i_* represents the mean pixel intensity within the ROI inside the MD inclusion post vaporization and μ_0_ corresponds to the mean pixel intensity within the ROI pre vaporization.

To investigate the expansion of the vaporized MDs, the MDs were imaged before and after vaporization using the Echo Revolution microscope with a 60× oil immersion objective (NA = 1.42). Their diameters were subsequently measured using the instrument’s accompanying analysis software.

### Numerical Modeling

The Marmottant model^41^ was employed to predict the vaporized MD expansion ratio. The maximum vaporized MD diameter was determined by multiplying the expansion ratio by the vaporized MD’s initial diameter. These predictions were then used to estimate the maximum pore diameter as a function of the PNP, and thus, to determine the applied PNP. Since the mean diameter of the fabricated MDs was ∼1.1 μm, the simulation was performed for a bubble with an initial diameter of 5.5 μm, based on the commonly reported 5-fold expansion upon vaporization.^42,43^ For comparison, simulations were also performed for a vaporized MD with a diameter of 1.1 μm to assess its behavior at the initial size. The stimulation was implemented in MATLAB. The Marmottant model is widely recognized for its strong alignment with experimental observation.^22,44^ This model takes into consideration parameters such as MB composition, the MB’s surrounding medium viscosity, and parameters related to the US excitation wave. Theoretical predictions for the vaporized MD expansion ratio were generated as a function of varying PNP (0–1000 kPa) at a center frequency of 200 kHz. The parameters were identical to those in ref.^22^ The surface tension of the vaporized MD outer radius was set to 0.073 N/m (saline) and to 0.04 for the inner radius. The shell density was 1000 kg/m^3^, the shell shear modulus was 122 MPa, and the shell viscosity was 2.5 Pa·s, the shell surface dilatational viscosity was 7.2 × 10^9^ N, and the elastic compression modulus was 0.55 N/m. The shell thickness was set to 1.55 nm.

### Collagen Scaffold Preparation

A solution was prepared by mixing Collagen I, Rat Tail (A10483–01, Gibco, Thermo Scientific, MA, USA) at an initial concentration of 3 or 4 mg/mL, 10× PBS (70013016, Gibco, Thermo Scientific, MA, USA), and 1 M sodium hydroxide solution (NaOH, S2770, Sigma-Aldrich, Milwaukee, WI) diluted in distilled water (15230089, Thermo Scientific, MA, USA) at a 1:4 ratio. The entire preparation process was conducted on ice to prevent premature collagen polymerization. The final collagen concentration was adjusted to 2.5 mg/mL, with PBS comprising 10% of the total mixture volume. NaOH was added incrementally until the pH reached a range of 6.5–7.5, as determined by the addition of phenol red solution (P0290, Sigma-Aldrich, Milwaukee, WI) to the PBS as a pH indicator. If needed, distilled water was added to reach the desired final volume. For collagen scaffold formation, MDs were added to the collagen solution at volumes of 10 or 0.5% relative to the solution, depending on the application: 10% for porosity and rheological studies, and 0.5% for in vitro experiments. A 300-μL aliquot of the mixture was then transferred into a circular mold (diameter: 1 cm, height: ∼3 mm) and allowed to partially polymerize at room temperature. The scaffolds were subsequently incubated at 37 °C in a humidified incubator for 30 min to achieve complete polymerization. After polymerization, the scaffolds were transferred to the US setup. The imaging and therapeutic transducers were operated simultaneously for 1 min. The imaging transducer transmitted a 2-cycle sinusoidal pulse at a PNP of 2.8 MPa (MI of 1.84), the optimized pressure, at a PRF of 20 Hz to vaporize the MDs. The low-frequency therapeutic transducer transmitted an 800 kPa (MI of 1.8) pulse, as determined by the Marmottant model, with a pulse length of 0.5 ms and a PRF of 33.33 Hz.

### Environmental Scanning Electron Microscopy

Environmental scanning electron microscopy (ESEM) was conducted using a Quanta 200 FEG ESEM (ThermoFisher, MA, USA) in high vacuum mode, with a working distance (WD) of 8.7–10.8 mm and an accelerating voltage of 5 and 10 kV. Imaging was performed to assess the porous structure of dual US-treated collagen scaffolds, containing 10% (v/v) MDs (MDs + Dual US), compared to nontreated collagen-only scaffolds (Collagen only) and dual US-treated collagen-only scaffolds (US only), and to determine the mean pore diameter. Additional control experiments were performed to isolate the individual contributions of vaporization and low-frequency insonation. Collagen hydrogels containing 10% MDs were exposed either to the 3.5 MHz rotating imaging transducer (Vaporization only, ADV-dominant condition) or to the 200 kHz therapeutic transducer (Low-frequency only). Prior to imaging, the samples were immersed in liquid nitrogen and freeze-dried using a lyophilizer (FDL-10N-50-TD-MM, MRC Lab, Israel) under conditions of temperature below −40 °C and vacuum pressure below 300 bar. To image the cross-section of the collagen scaffolds, the freeze-dried samples were immersed in liquid nitrogen for approximately 1 min and then fractured to expose the internal collagen structure. Subsequently, all samples were sputter-coated with a gold/palladium layer. The acquired ESEM images were analyzed using ImageJ software to quantify the average pore diameter within the scaffolds.

### Collagen Scaffold Porosity Study

These experiments were conducted to compare the structure of dual US-treated collagen scaffolds containing 10% MDs (v/v) with nontreated collagen-only scaffolds. The scaffolds were harvested, cryo-sectioned into 5-μm-thick slices and stained with hematoxylin (Leica 3801542) following a standard protocol. The slides were scanned using the Echo Revolution microscope at 4× and 20× optical magnification. To compare and quantify the porosity of the slices, postprocessing of the scanned images was carried out using ImageJ. Each image was converted to black and white by applying a threshold, such that the pore regions appeared white, and the dense regions appeared black. Porosity [%] was then calculated as the ratio of white pixels to the total number of pixels, multiplied by 100, as shown in (eq 2)­

2 $$porosity\left[\%\right]=\frac{numberofwhitepixels}{totalnumberofpixels}\times 100\left[\%\right]$$

### Rheological Analysis

The rheological properties of the collagen scaffolds were analyzed using an ARES-G2 rheometer (TA Instruments, New Castle, DE, USA) with an 8 mm parallel-plate geometry. This analysis was performed to evaluate the differences in rheological properties between dual US-treated collagen scaffolds containing 10% (v/v) of MDs (MDs + Dual US), nontreated collagen scaffolds containing 10% (v/v) MDs (MDs only), and nontreated collagen-only scaffolds (Collagen only). A gap ranging between 600 and 700 μm was set to ensure proper contact between the geometry and the samples. Oscillatory strain tests (0.01–100%) were conducted at a frequency of 1 Hz and a temperature of 25 °C to evaluate the storage modulus (*G*’), loss modulus (*G*’’), loss tangent (tan δ = *G*’’/*G*’), and complex viscosity (η*). To ensure reproducibility, measurements were performed on at least three independent samples.

### Cell Culture Experiments

These experiments were designed to assess the migration and proliferation capacity of cells within dual US-treated collagen scaffolds containing 0.5% (v/v) of MDs and nontreated collagen-only scaffolds (Collagen only). Mouse fibroblast cells were genetically modified to express green fluorescent protein (GFP) protein. The GFP-expressing fibroblasts were cultured in Dulbecco’s Modified Eagle Medium (DMEM, high glucose, with l-glutamine), supplemented with 10% (v/v) fetal bovine serum, 1% (v/v) penicillin–streptomycin, 0.11 g/L sodium pyruvate, and 6 μg/mL puromycin. Cells were maintained at 37 °C in a humidified incubator with 5% CO_2_ until they reached approximately 85% confluency on the day of seeding. Cells were then dissociated using trypsin, resuspended at a concentration of 2 × 10^6^ cells in 50 μL of medium, and embedded within the scaffolds placed in a 6-well plate, based on a previously reported protocol.^45^ 2.5 mL of the cell culturing medium was added to each well. The plate was then incubated at 37 °C in a humidified 5% CO_2_ incubator for 1 week. The medium was replaced every 2–3 days. After 1 week, the scaffolds were harvested, cryo-sectioned into 5-μm-thick slices and stained with hematoxylin and eosin (Leica 3801602) (H&E) following a standard protocol. The H&E slides were scanned using the Echo Revolution microscope at 4× and 20× optical magnification. Additionally, cell viability within the scaffolds was assessed after 1 week using the fluorescent dye 7-Aminoactinomycin D (7-AAD; Thermo Fisher Scientific, A1310) to detect dead cells. The 7-AAD was added to the culture medium at a final concentration of 5 μg/mL and incubated for 15 min. Following incubation, the medium was replaced, and the scaffolds were rinsed with PBS prior to microscopic imaging. For quantitative analysis, the scaffolds were enzymatically digested using a collagenase solution (1 mg/mL collagenase in 5 mM CaCl_2_). After 1 week of incubation, the scaffolds were rinsed twice with PBS, and 300 μL of the collagenase (C2674, Sigma-Aldrich, Milwaukee, WI) solution was added to each scaffold, followed by incubation. After 1 h, the solution was pipetted out and returned to the incubator for an additional 30 min. At this point, the scaffolds were fully digested. The cells were then pelleted and resuspended in fresh medium. The number of cells within each scaffold was quantified using a cell counting instrument (CellDrop, DeNovix Inc., Wilmington, USA). For the quantitative analysis, four groups were tested: nontreated collagen-only (Collagen only), dual US-treated collagen-only scaffolds (US only), nontreated collagen scaffolds containing 0.5% (v/v) of MDs (MDs only), and dual US-treated collagen scaffolds containing 0.5% (v/v) of MDs (MDs + Dual US) (*n* = 10 per group).

### Statistics

Statistical analyses were performed using Prism 9 (GraphPad Software, Inc.). Data are presented as mean ± standard deviation (SD). Normality was assessed using the Shapiro-Wilk test. For data sets that deviated from normality, the nonparametric Kruskal–Wallis test was applied. For normally distributed data, one-way ANOVA was used. A p-value of less than 0.05 was considered statistically significant. All experiments were conducted in triplicate or quadruplicate, as specified per experiment.

## Results and Discussion

### Microdroplet Characterization

The average diameter of the lipid-shell, PFP liquid-core MDs was 1.03 ± 1.15, with a concentration of 4.8 × 10^6^ particles mL^–1^ (Figure 2A). The relatively high standard deviation is attributed to the use of a herringbone mixer-based microfluidic chip, which promotes efficient mixing but typically produces broader size distributions compared to T-junction or flow-focusing devices. Notably, such variability is common for droplet and US contrast agent populations.^46,47^ Microscopy confirmed spherical MD morphology and diameters consistent with particle sizing measurements (Figure 2B). Fluorescence microscopy further verified the formation of fluorescent MDs with a similar size distribution to blank MDs (Figure 2C). After 7 months of storage, the measured concentration was 1.62 × 10^8^ particles mL^–1^, and the mean diameter was 0.8 ± 0.43 μm (Figure S1).

**Figure 2:**
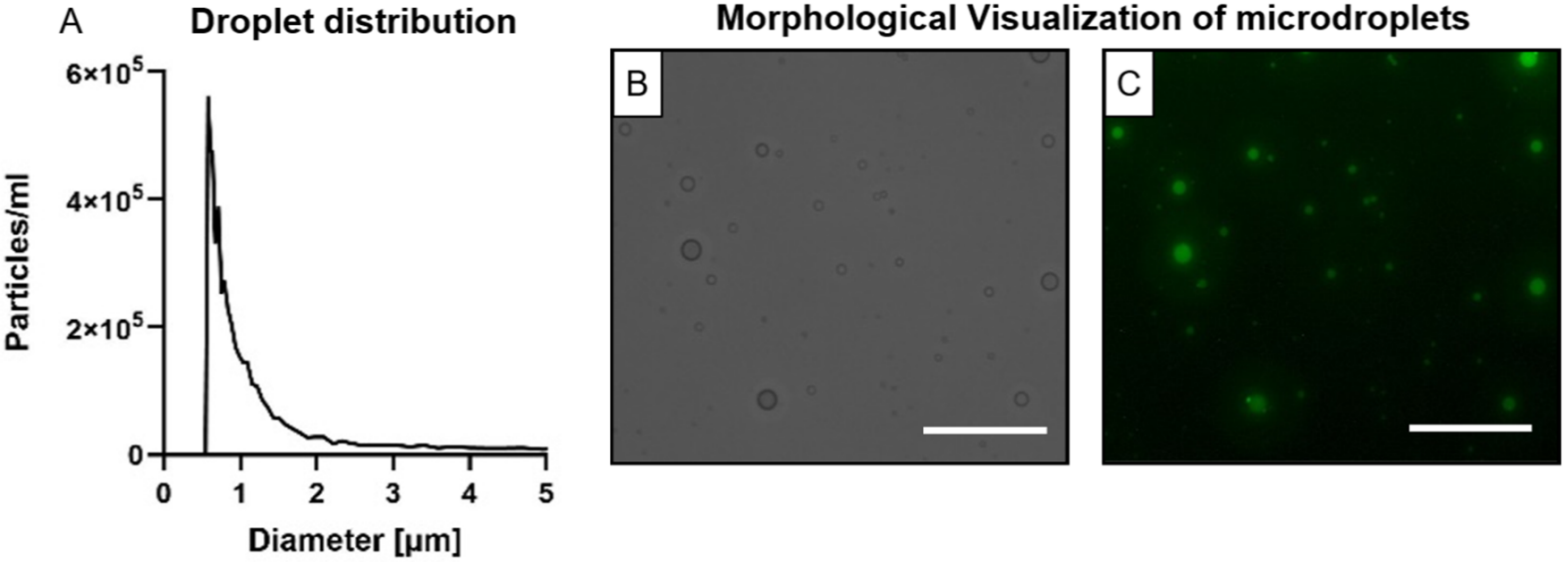
MDs characterization. (A) MD size distribution. (B) Bright field microscope image of the MDs. (C) Fluorescence microscope image of the fluorescent MDs. Scale Bar: 50 μm.

### Microdroplet Stability and Vaporization Pressure Optimization

The temporal stability of the MDs without US exposure was evaluated at 37 °C to mimic physiological conditions. A suspension of MDs diluted in degassed PBS was injected into the rod inclusion of an agar phantom, and contrast changes were monitored. In their liquid state, MD-filled inclusions appeared hypoechoic, whereas vaporization produced hyperechoic contrast due to MB formation. Therefore, an increase in inclusion contrast was considered indicative of MD vaporization. No significant contrast change was observed over 1 h at 37 °C, indicating that the MDs remained stable during collagen polymerization (*p* ≥ 0.05, Figure 3A).

**Figure 3:**
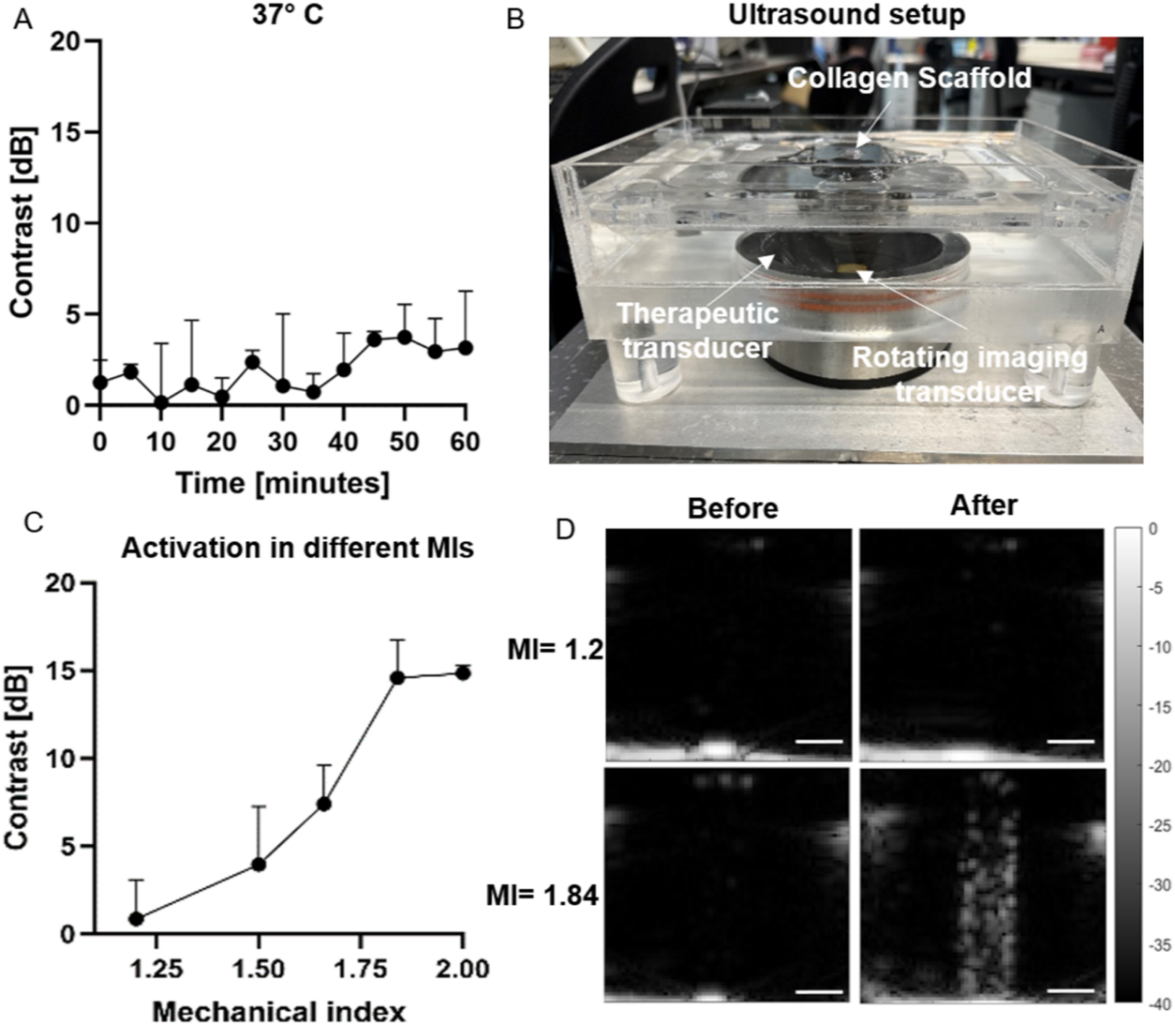
MD stability and vaporization optimization results. (A) Contrast inclusion as a function of time at 37 °C. (B) US setup. (C) Contrast enhancement following MD ADV as a function of the MI. (D) US images of the MD-filled inclusion before and after US vaporization. All experiments were performed in triplicate. Data are presented as mean ± SD. Scale bar: 5 mm.

Active vaporization was then optimized using an US-guided focused US (USgFUS) system composed of an imaging array integrated within a therapeutic array, enabling volumetric MD vaporization (Figure 3B). Following vaporization, the inclusion became hyperechoic, and the contrast pre- and post vaporization was calculated.

Vaporization was evaluated at PNPs ranging from 2.3 to 3.8 MPa, corresponding to MI values of 1.2–2.0. Contrast increased with MI but plateaued above MI of 1.84 (Figure 3C). A PNP of 3.4 MPa (3.5 MHz, MI = 1.84) produced the highest contrast enhancement (14.6 dB) while remaining within the FDA safety limit (MI < 1.9),^48^ which correlates with a higher concentration of vaporized MDs and is therefore expected to promote greater pore formation within the scaffold. Consequently, this pressure was selected for subsequent experiments. Representative US images before and after vaporization are shown in Figure 3D. To quantify MD expansion following vaporization, MDs were imaged using a microscope before and after insonation. Prior to insonation, the mean MD diameter was 1.26 ± 0.47 μm, which increased to 6.38 ± 1.28 μm after insonation, corresponding to an average expansion ratio of 5.05 (*n* = 32, *p* < 0.0001; Figure S2). This result is consistent with the commonly assumed ∼5-fold increase in droplet diameter upon vaporization.^42,43^ More broadly, the choice of MD core material influences stability and vaporization efficiency: compounds with higher boiling points (e.g., perfluorohexane, perfluorooctane) enhance stability but require higher acoustic pressures for vaporization. PFP was selected here as a compromise between stability and efficiency, yet it could be adjusted depending on the application.

### Marmottant Model Prediction

Low-frequency US at a center frequency of 200 kHz was then applied to the vaporized MDs to induce their collapse. Numerical simulations based on the Marmottant model were performed to estimate the maximum diameter of vaporized MDs under these conditions and guide parameter selection for the therapeutic transducer. Simulations were conducted for vaporized MDs with resting diameters of 1.1 μm, as a lower bound, and 5.5 μm, assuming a 5-fold expansion following ADV. The expansion factor during ADV depends on multiple parameters, including droplet formulation, surrounding medium properties, and temperature. Here, a factor of 5 is used as a conservative, order-of-magnitude estimate that may vary under different conditions. The predicted maximum vaporized MD diameter served as an estimate for the resulting pore size as a function of PNP (Figure 4A). To achieve the largest possible pores while remaining within FDA safety limits, a PNP of 800 kPa (200 kHz, MI = 1.8) was selected, corresponding to a predicted maximal diameter of ∼56 μm. It is interesting to note that, although the predicted maximal diameter is relatively similar for initial diameters of 1.1 and 5.5 μm, and becomes nearly identical at 1000 kPa, the calculated expansion ratio, is significantly higher for the 1.1 μm vaporized MD. For an MI of 1.8, the predicted expansion ratio is 10 for a 5.5 μm MB and 44 for a 1.1 μm initial diameter. While expansions greater than ∼3.5× are often associated with inertial cavitation, high-speed imaging studies have reported bubble expansions up to ∼35× at similar frequencies, supporting the physical plausibility of these predictions.^22^

**Figure 4:**
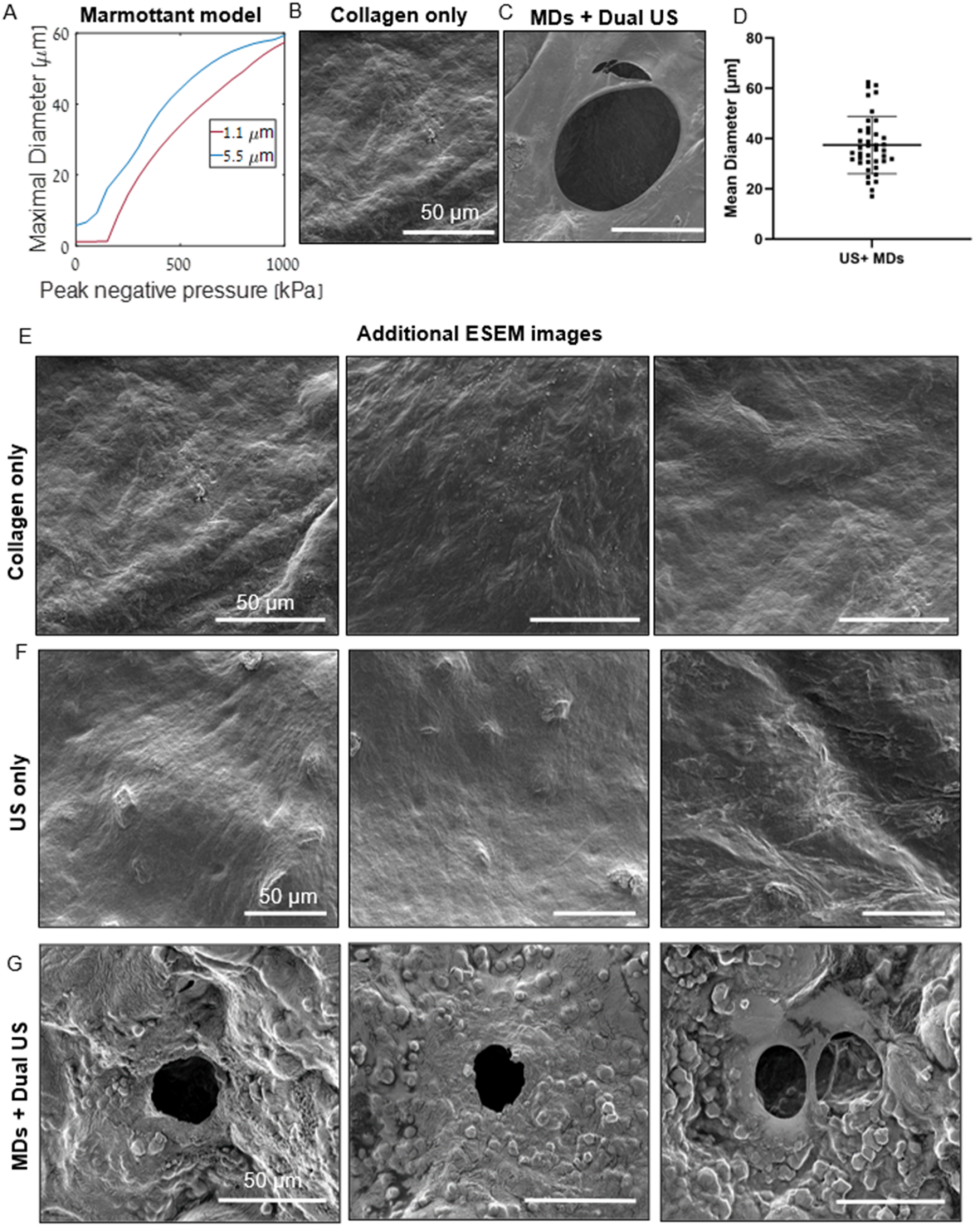
Micropore characterization. (A) Theoretical predictions of the maximal diameter as a function of PNP at a center frequency of 200 kHz for a MB and MD with initial radii of 2.75 and 0.55 μm, respectively. Representative ESEM images of (B) Collagen only and (C) MDs + Dual US scaffolds. (D) The generated pores diameter distribution. (E–G) Additional representative ESEM images. (E) Collagen only scaffolds. (F) US only scaffolds. (G) MDs + Dual US scaffolds. Scale bar: 50 μm.

The Marmottant model was used here to approximate the expansion behavior of vaporized MDs by assuming dynamics similar to those of MBs. However, the actual diameter of vaporized MDs within the collagen scaffold may be smaller than predicted, as the fibrillar structure, stiffness, and increased viscosity of the hydrogel can mechanically constrain MD expansion during vaporization. Similar reductions in ADV-generated bubble size have previously been observed in confined or viscous biological media such as blood plasma compared to PBS.^49,50^ Accordingly, the predicted values should be considered an upper bound. More detailed modeling of these environmental effects could be pursued in future studies using finite element approaches (e.g., COMSOL), enabling simulation of bubble dynamics under low-frequency insonation in media with properties matching those of collagen hydrogels. In addition, standing waves generated by the US setup may introduce spatial variations in acoustic pressure, potentially affecting local ADV efficiency and subsequent bubble oscillation.^51^ Nevertheless, the observed pore formation was reproducible across replicate samples. To experimentally verify MD destruction under these conditions, the optimized USgFUS setup was used. After MD vaporization, the 200 kHz therapeutic transducer delivered a pulse at 800 kPa (MI = 1.8). US imaging confirmed that the signal from vaporized MDs disappeared and the inclusion returned to a hypoechoic state, indicating droplet collapse (Figure S3).

### Collagen Scaffold Porosity

Next, the MDs were embedded within a collagen scaffold and subjected to dual-frequency US insonation consisting of vaporization by a rotating transducer at 3.5 MHz and collapse induced by a 200 kHz therapeutic transducer. Although the 200 kHz transducer has a relatively large focal volume due to its low frequency, localization is achieved via dual-frequency excitation: the imaging transducer is tightly focused on the collagen hydrogel plane and spatially gates vaporization. The predicted MB expansion was then correlated with the experimentally observed pore sizes within the scaffold. Scaffold structure was evaluated by ESEM and histology for MDs + Dual US, Collagen only, and US only groups. ESEM images of the Collagen only and US only scaffolds revealed smooth surfaces (Figure 4B,E,F), indicating that US exposure without MDs did not generate pores. To further isolate the contribution of each US frequency component, additional control experiments were performed in which MD-loaded scaffolds were exposed to either the imaging transducer alone (ADV-dominant condition) or the low-frequency transducer alone. Both Vaporization only and Low-frequency only groups similarly exhibited smooth scaffold surfaces without observable pores (Figure S4A,B), demonstrating that pore formation requires the combined action of both frequency components and highlighting the importance of the dual-frequency insonation protocol. In contrast, the MDs + Dual US group exhibited a porous structure (Figures 4C,G, and S4C). The spherical structures observed in the MDs + Dual US images are attributed to salt residues originating from PBS used during MD and scaffold preparation. Higher-magnification imaging further revealed the underlying fibrillar architecture of the collagen scaffold (Figure S5). The average pore size in the MDs + Dual US group was 39.42 ± 10.28 μm (Figure 4D), slightly smaller than predicted by the theoretical model, likely due to the mechanical constraints imposed by the collagen microstructure. The observed pore size distribution reflected that of the precursor MDs (0.6–1.3 μm), consistent with the size-dependent nature of ADV and subsequent bubble dynamics. Notably, the resulting pores fall within the range reported to support dermal and epidermal regeneration,^52^ and have been associated with favorable healing outcomes and reduced foreign body responses,^53^ suggesting potential suitability of the scaffold for such applications. Importantly, pore characteristics can theoretically be tailored by adjusting MD size through microfluidic parameters such as total flow rate, flow rate ratio, and channel geometry. Pore dimensions may also be modulated by varying the acoustic pressure, enabling customization for specific tissue engineering applications.

Fluorescence microscopy images of the scaffolds showed minimal signal prior to US exposure, whereas a marked increase in fluorescence was observed after insonation, indicating release of the fluorescent payload from the MDs into the scaffold (Figure 5A), concurrent with pore formation. Looking ahead, this platform also holds considerable promise for controlled release applications. Encapsulating growth factors such as VEGF or fibroblast growth factors (aFGF, bFGF) within MDs could enhance neovascularization, thereby improving oxygen and nutrient delivery to regenerating tissues.^34,54–56^ Similarly, antibiotics or other therapeutic agents could be incorporated to enable spatiotemporally controlled release upon US vaporization, reducing adverse biological responses and broadening clinical utility.^57^

**Figure 5:**
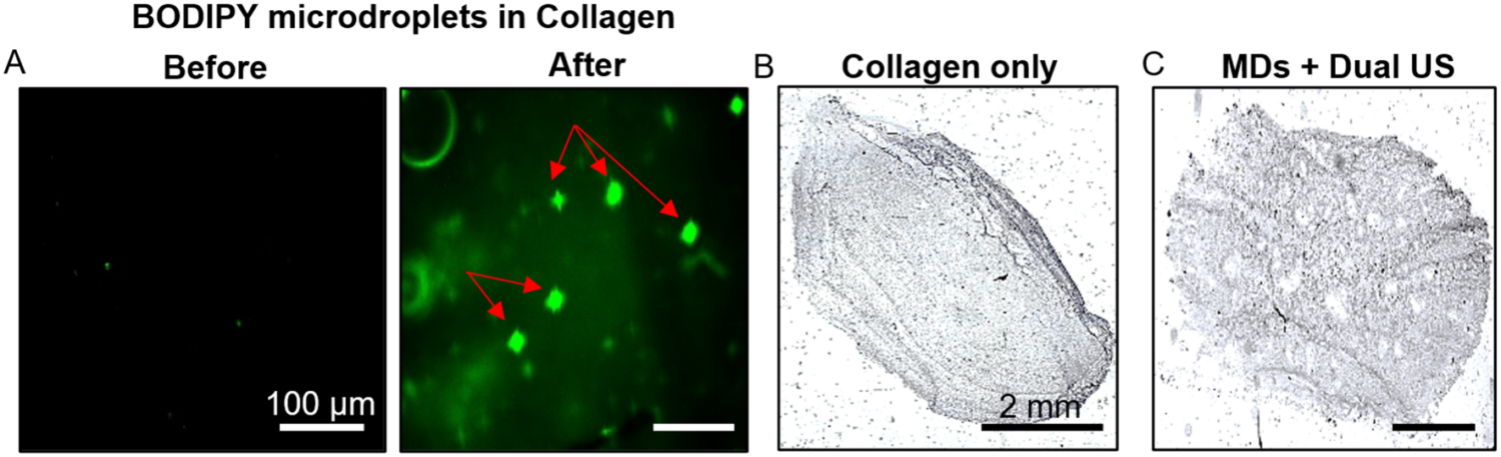
Fluorescence MD vaporization and porous scaffold imaging. (A) Microscope images of collagen with MDs, before and after US exposure. Red arrows indicate pores formed in the collagen scaffold following insonation. Scale bar: 100 μm. (B, C) H&E histopathology imaging of 5 μm sections of (B) Collagen only, and (C) MDs + Dual US scaffold. (B, C) Scale bar: 2 mm.

Microscopic analysis of 5 μm-thick scaffold sections further revealed a porous structure in the MDs + dual US group, with a porosity of 56.53 ± 3.91%, compared to 27.73 ± 2.84% in Collagen only scaffolds (*p* < 0.0001; Figure 5B,C). Unvaporized MDs (mean diameter ∼1.1 μm) are below the resolution of histological sections and are therefore unlikely to appear as pores or significantly affect porosity measurements. Accordingly, porosity was not quantified for MD-containing scaffolds without US, and the observed increase is attributed primarily to US-induced ADV. Scaffold porosity is influenced by the concentration of embedded MDs; however, a current limitation is the inability to spatially vary MD concentration within a single scaffold, which restricts the creation of gradients or region-specific effects. Taken together, these results indicate that both surface and internal structural changes arise from the mechanical effects associated with the collapse of vaporized MDs.

### Rheological Analysis

Beyond structural characteristics, the mechanical properties of scaffolds play a critical role in regulating cellular behavior and supporting functional tissue regeneration. Accordingly, the mechanical properties of the scaffolds were systematically evaluated by rheology to determine the influence of US treatment and MD incorporation on their viscoelastic behavior. Oscillatory strain sweep tests (0.01%–100%, 1 Hz, 25 °C) were performed on three scaffold types: Collagen only, collagen with embedded MDs (MDs only), and dual US-treated scaffolds containing MDs (MDs + Dual US). The parameters assessed included storage modulus (*G*′), loss modulus (*G*″), damping factor (tan δ), and complex viscosity (η*). *G*′, indicative of the scaffold’s elastic response and stiffness,^58^ remained constant at low strain levels but exhibited a pronounced decline beyond the critical strain in all groups. Among them, MDs + Dual US displayed the highest initial *G*′, while MDs only showed the lowest (Figure 6A, *p* < 0.01). One possible explanation for this difference is that incorporation of MDs into the collagen solution reduces the effective collagen concentration, which may in turn affect scaffold mechanical integrity. The observed increase in elasticity is attributed to compaction occurring during MD vaporization.^35^ Rapid volumetric expansion generates local stresses that compress and densify collagen fibrils, enhance packing and effective cross-linking. Although porous scaffolds typically have reduced mechanical strength, compaction here offsets this effect, enabling both pore formation and increased scaffold elasticity. However, the presence of residual MBs may also contribute to the measured mechanical response, potentially leading to an apparent stiffening effect. Similarly, *G*″, representing the viscous component and energy dissipation capacity,^58^ followed a comparable trend. The highest initial *G*″ values were observed in the MDs + Dual US scaffolds, and the lowest in MDs only group, with *G*″ decreasing moderately as strain increased (Figure 6B, *p* < 0.0001). The damping factor, tan­(δ), which expresses the ratio of viscous to elastic behavior, began at comparable low values (∼0–0.05) across all samples, indicating a predominantly elastic nature. With increasing strain, tan­(δ) exhibited a steep increase, denoting a transition toward more viscous behavior. The MDs only scaffolds reached the highest final tan­(δ) values, while the MDs + Dual US group remained the lowest (Figure 6C, not significant), meaning that MD + Dual US best preserved their elastic behavior under mechanical stress and demonstrated the greatest resistance to deformation. η*, representing overall resistance to deformation, mirrored the trend of *G*′. It remained stable at low strain but decreased sharply after the critical strain point. MDs + Dual US scaffolds demonstrated the highest initial η*, whereas the lowest values were observed in MDs only group (Figure 6D, *p* < 0.01).

**Figure 6:**
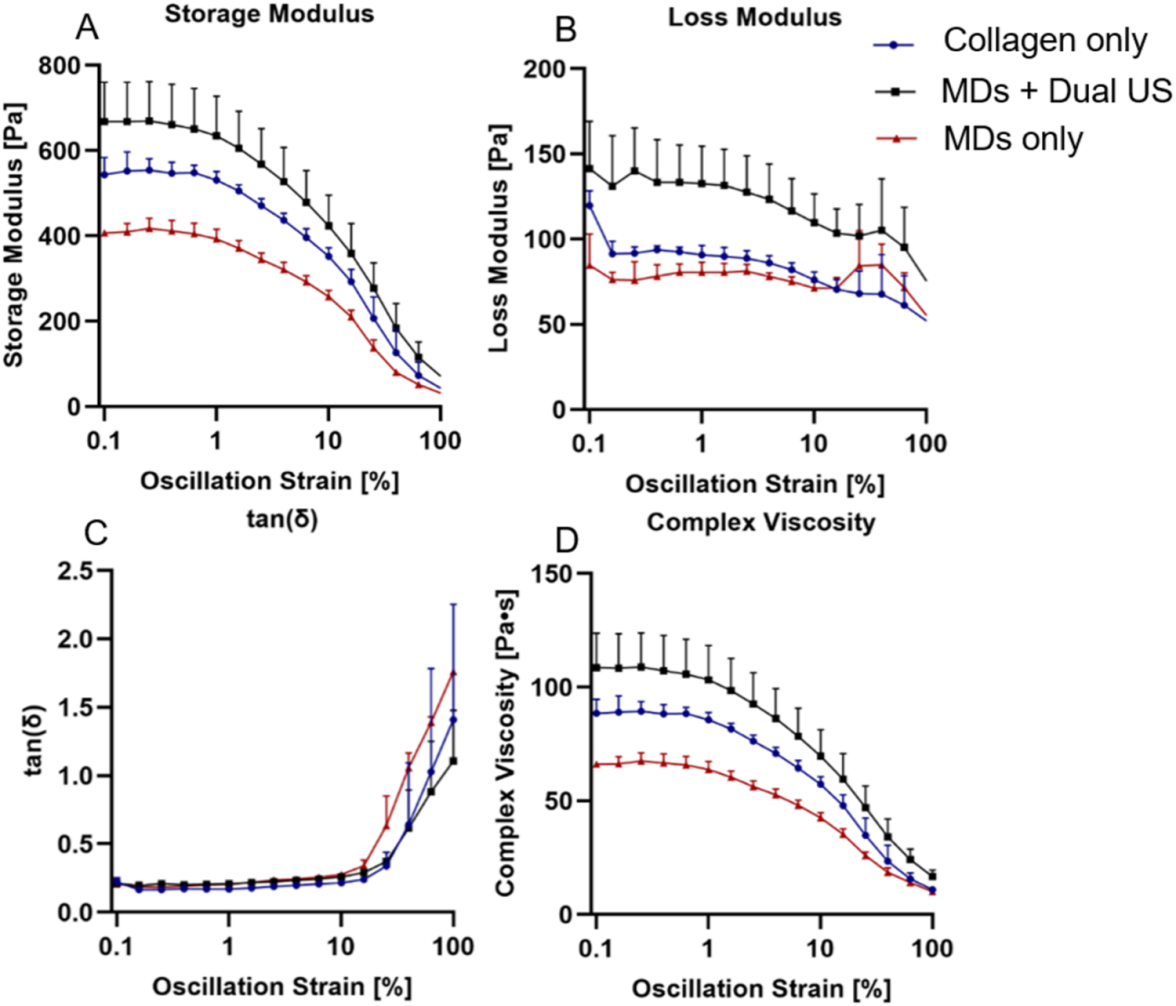
Rheological analysis of the scaffolds. Compared groups include Collagen only (blue line), MDs + Dual US (black line), and MDs only groups (red line). (A) Storage modulus (*G*′), (B) Loss modulus (*G*″), (C) Damping factor (tan­(δ)), and (D) Complex viscosity (η*) as a function of oscillation strain. All experiments were performed in triplicate, and data are presented as mean ± SD.

The observed US-mediated modulation of scaffold mechanics highlights the potential of this platform for applications requiring dynamic tuning of material properties over time. Specifically, the ability to alter scaffold stiffness following fabrication may be advantageous in regenerative settings where softer matrices are initially preferred to promote cell migration and attachment, while increased stiffness at later stages may provide improved mechanical support to regenerating tissue, as cell attachment is known to decrease with increasing substrate stiffness.^59^ In addition, stiffer scaffolds may be used to direct mesenchymal stromal cell (MSC) differentiation, consistent with previous reports showing that acoustic vaporization-induced stiffening of collagen matrices promoted osteogenic marker expression in MSCs.^35^ Thus, the present approach may offer a means to temporally regulate scaffold mechanics for guided cell differentiation and tissue maturation. Beyond collagen-based systems, this strategy could potentially be extended to other acoustically responsive biomaterials, including US-sensitive polymers capable of undergoing polymerization upon insonation.^60^ Incorporating MDs into such materials may enable simultaneous US-triggered polymerization and pore formation, thereby expanding the versatility of the platform for broader tissue engineering applications.^59^

### In Vitro Cell Viability on the Scaffold’s Experiments

To assess the effect of the MDs + Dual US treatment on cell viability within the scaffold, fibroblast cells mixed with MDs were seeded into the scaffold, treated with US, and evaluated 1 week post-treatment. For the Collagen only and MDs + Dual US scaffolds, cells were distributed throughout the matrix (Figure 7A,C). As indicated by 7-AAD staining and GFP expression, the majority of the cells were viable, with only a few exhibiting signs of cell death (Figure 7B,D), indicating that the materials were nontoxic over the incubation period. To quantify cell numbers within each scaffold, samples were enzymatically digested using collagenase. Comparisons were made between NTC (Collagen only), US only, MDs only and the MDs + Dual US scaffolds. The values above 100% are normalized to the Collagen only group 7 days post cell seeding. The MDs + Dual US group exhibited the highest cell count among all experimental conditions, showing a 28% increase relative to the Collagen only control (*p* < 0.001), a 25% increase relative to the US only group (*p* < 0.005), and a 33% increase relative to the MDs only group (*p* < 0.0001) (Figure 7E). The significant increase in the MDs + Dual US group highlights the role of generated pores in supporting cell viability by improving nutrient and oxygen transport and waste removal. In future studies, in vivo experiments will be used to further validate these findings.

**Figure 7:**
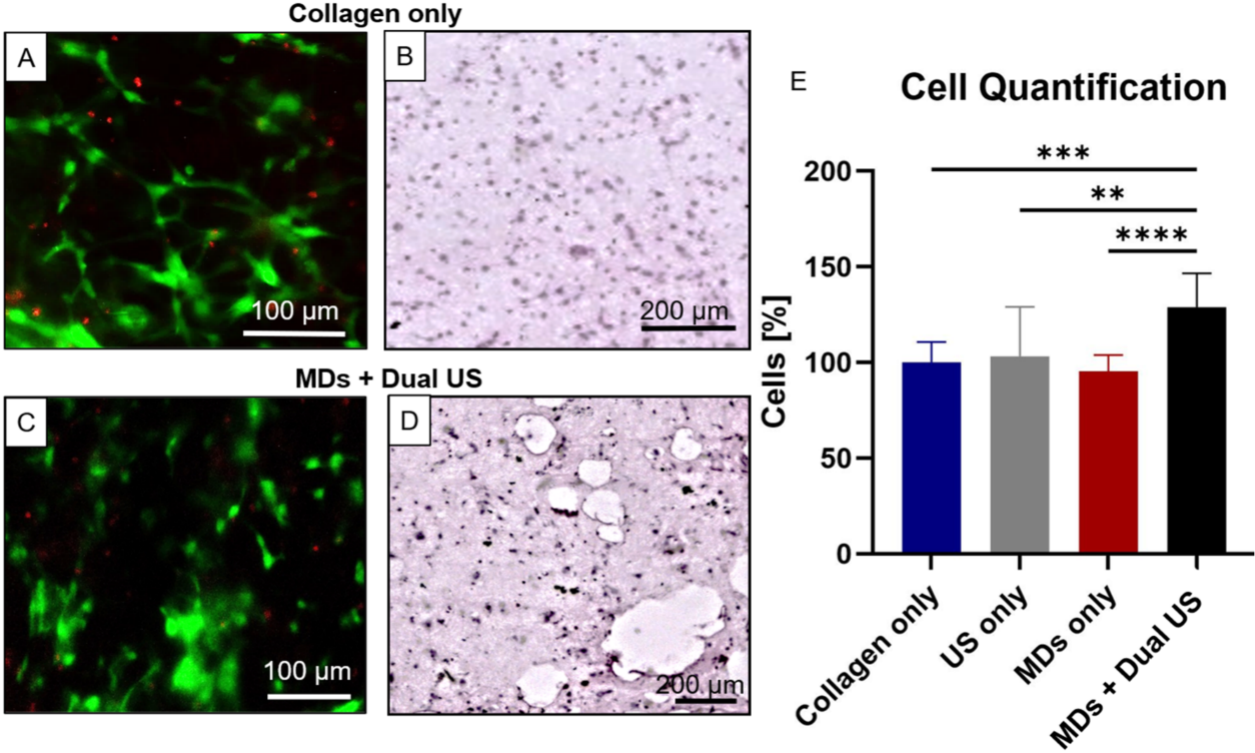
Cell viability and proliferation in response to US and MDs in vitro. Fluorescent microscopy images of fibroblasts embedded in (A) Collagen only and (C) MDs + Dual US scaffolds, showing live cells in green and dead cells in red. H&E-stained 5 μm section of fibroblasts in a (B) Collagen only, and a (D) MDs+ Dual US scaffolds. (E) Cell quantification for Collagen only, US only, MDs only and MDs + Dual US scaffolds. One-way ANOVA with Tukey’s multiple comparison test (*N* = 10). Adjusted p values were **p < 0.005, ***p < 0.001, ****p < 0.0001.

## Conclusions

In this study, we developed a dual-frequency US approach for noninvasive generation of porous collagen scaffolds using phospholipid-coated perfluoropentane MDs as acoustically activatable porogens. By combining MHz-frequency ADV with subsequent low-frequency cavitation, this strategy enabled controlled pore formation through a two-step activation mechanism while maintaining US exposures within clinically relevant MI limits. This approach reduces the pressure required for pore generation compared with conventional single-frequency droplet activation methods. Compared with traditional porous scaffold fabrication techniques, the proposed method offers several advantages, including elimination of toxic porogen residues, tunable and spatiotemporally controlled pore generation, and simultaneous modulation of scaffold mechanical properties following fabrication. The use of thermally stable lipid-coated MDs further improves stability compared with conventional MBs-based approaches. Using this platform, we generated porous collagen scaffolds with pore sizes in the tens-of-micrometers range, significantly increased scaffold porosity, and enhanced viscoelastic properties despite pore formation. The resulting scaffolds supported high fibroblast viability and increased cell proliferation, indicating that the generated porous microarchitecture is biologically favorable. Overall, this work establishes dual-frequency US-mediated activation of MDs as a promising strategy for tunable scaffold engineering and controlled modulation of biomaterial structure and mechanics for tissue engineering applications.

## Supplementary Material



## Data Availability

The data sets generated during and/or analyzed during the current study will be made available upon request.

## Notes

Comparison of MD size distributions for fresh and 7-month-old samples (Figure S1); experimental measurement of MD expansion following vaporization using optical microscopy (Figure S2); US images of MDs in a gelatin phantom (Figure S3); individual US component contributions assessed by ESEM imaging (Figure S4); ESEM images of the collagen scaffold cross-section (Figure S5) (PDF)
