# Supplementary material for: Dual-Frequency Ultrasound Enhances Cavitation of Microdroplets for Controlled Scaffold Porosity in Tissue Engineering
Source: ACS Appl Mater Interfaces. 2026 Apr 30;18(18):25778–90. doi: 10.1021/acsami.5c19957 (PMC13181721; doi:10.1021/acsami.5c19957)
Supplement: Supplementary file 1 [file am5c19957_si_001.pdf]

Supplementary Information

Dual-frequency ultrasound enhances cavitation of microdroplets for controlled scaffold porosity in tissue engineering

Hen Shenhav[a], Bar Glickstein[a], Tiran Bercovici[a], Offir Loboda[b,c,d,e], Gal Shklarski Shchori[a], Dekel Rosenfeld[a,c,f], Lihi Adler- Abramovich[b,c,d] and Tali Ilovitsh[a,c,d,f*]

[a] School of Biomedical Engineering, Iby and Aladar Fleischman Faculty of Engineering, Tel Aviv University, Tel Aviv, 6997801 Israel
[b] Department of Oral Biology, the Goldschleger School of Dental Medicine, Gray Faculty of Medical & Health Sciences, Tel Aviv University, Tel Aviv, 6997801 Israel
[c] Jan Koum Center for Nanoscience and Nanotechnology, Tel Aviv University, Tel Aviv, 6997801 Israel
[d] The Center for Physics & Chemistry of Living Systems, Tel Aviv University, Tel Aviv, 6997801 Israel
[e] Department of Materials Science and Engineering, Iby and Aladar Fleischman Faculty of Engineering, Tel Aviv University, Tel Aviv, 6997801 Israel
[f] The Sagol School of Neuroscience, Tel Aviv University, Tel Aviv, 6997801 Israel

* Corresponding author: ilovitsh@tauex.tau.ac.il

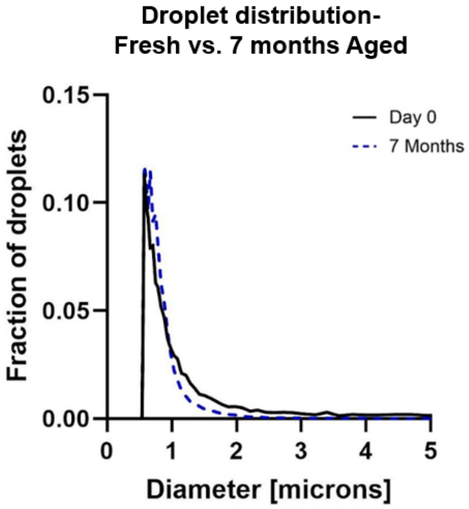

Fig. S1 Comparison of MD size distributions for fresh and 7-month-old samples. Fresh MDs (black) versus 7-month-old MDs (dashed blue).

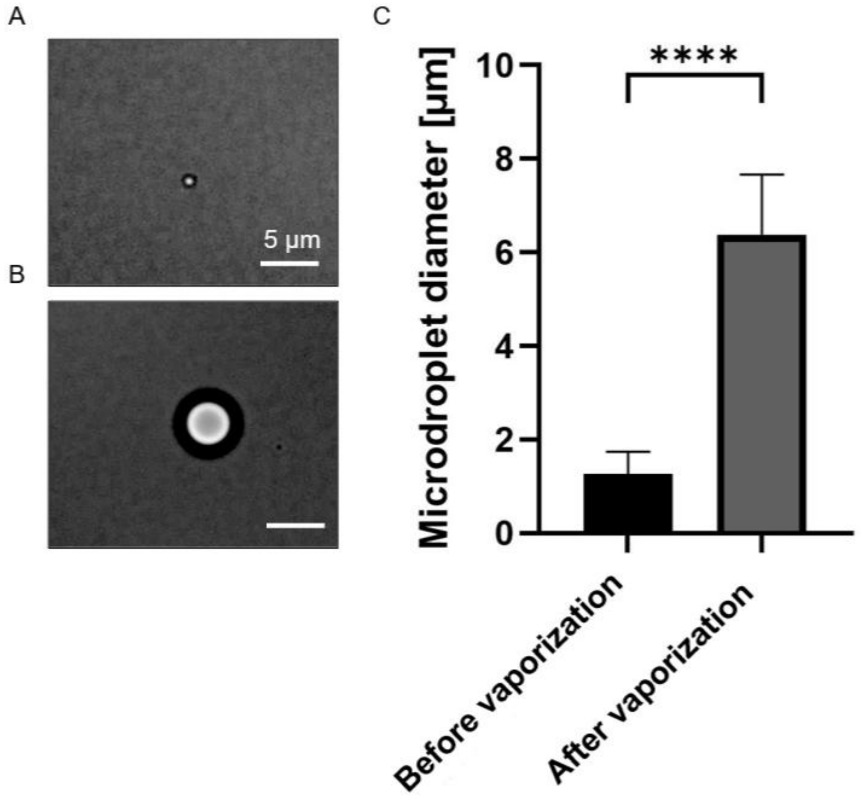

Fig. S2 Experimental measurement of MD expansion following vaporization using optical microscopy. (A) MDs before vaporization, and (B) after vaporization, imaged using an optical microscope with a 60× oil immersion objective (NA = 1.42). (C) MD diameter distribution before and after vaporization. Statistical analysis was performed using an unpaired t-test. ****$p < 0.0001$.

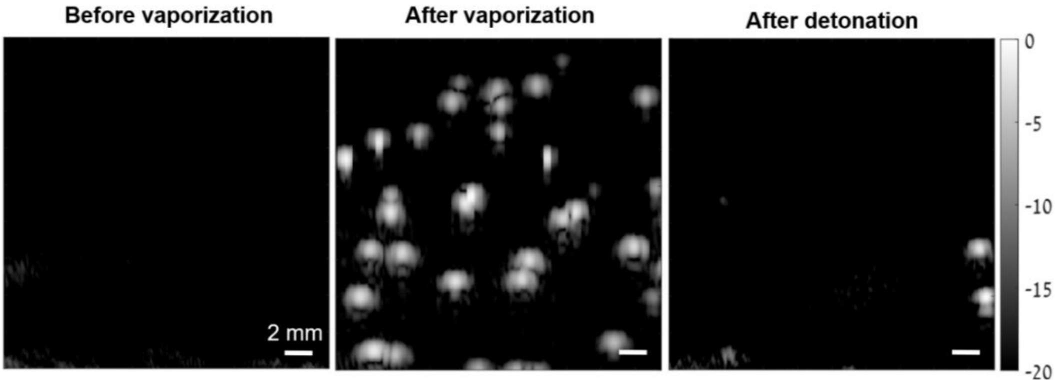

Fig. S3 US images of MDs in a gelatin phantom. Left: before vaporization, the gelatin appears hypoechoic. Middle: after vaporization, the gelatin becomes hyperechoic due to the presence of vaporized MDs. Right: after detonation, the gelatin returns to a hypoechoic appearance, indicating bubble implosion.

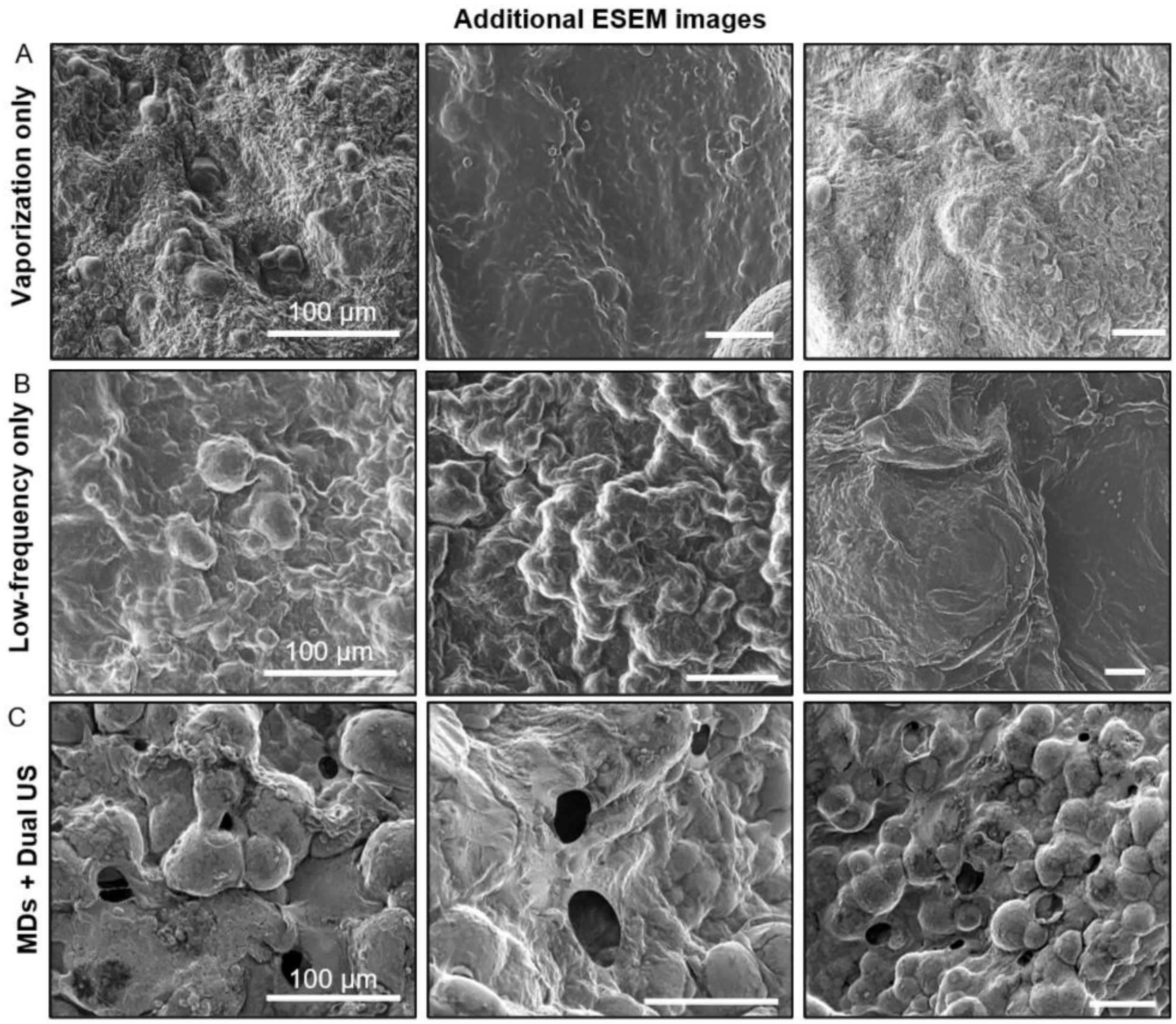

Fig. S4 Individual US component contributions assessed by ESEM imaging. (A) Vaporization only scaffolds. (B) Detonation only scaffolds. (C) MDs + Dual US scaffolds. Scale bar: 100 µm.

Collagen scaffold cross-section

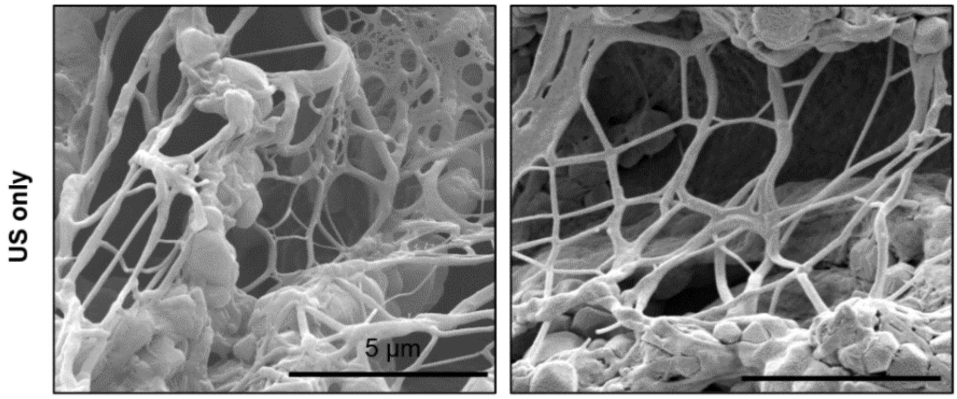

Fig. S5 ESEM images of the collagen scaffold cross-section, showing its fibrillar structure. Scale bar: 5 µm.
